# Utilization of pipeline embolization device for treatment of ruptured complex intracranial aneurysms

**DOI:** 10.3389/fneur.2025.1673718

**Published:** 2026-01-13

**Authors:** Yong-Feng Han, Qian Zhao, Xue-Jing Zhang, Dong-Liang Zhang, Lei Yang

**Affiliations:** 1 Department of Neurosurgery, Shijiazhuang People’s Hospital, Shijiazhuang, Hebei, China; 2 Emergency Medicine Department, Shijiazhuang Second Hospital, Shijiazhuang, Hebei, China; 3 Center of Medical Research, Shijiazhuang People’s Hospital, Shijiazhuang, Hebei, China

**Keywords:** pipeline embolization device, flow diversion, ruptured, complex intracranial aneurysms, antiplatelet therapy

## Abstract

**Objective:**

Use of the Pipeline embolization device (PED) for treatment of ruptured complex intracranial aneurysms (IAs) remains controversial due to higher thromboembolic and hemorrhagic complications compared to balloon-assisted coiling. We present our experience using the PED for ruptured complex IAs and focus on the safety, effectiveness, and follow-up results.

**Methods:**

Consecutive 46 patients with ruptured complex IAs who had undergone PED deployment from January 2019 to December 2023 at our neurosurgical center were retrospectively enrolled. Patient demographics, aneurysm characteristics, procedural complications, clinical and angiographic follow-up outcomes were reviewed.

**Results:**

A total of 46 patients were analyzed with a mean age of 55.8 ± 13.4 years, including 30(65.2%) females. WFNS grades were I in 27 patients (58.7%), II in 10 (21.7%), III in 5 (10.9%), IV in 2 (4.3%), and V in 2 (4.3%). The ruptured aneurysms included 12 (26.1%) saccular, 23 (50.0%) blister-like, 10 (21.7%) dissecting, and 1(2.2%) fusiform. The average size of IAs was 4.3 ± 2.9 mm. PED deployment was technically successful in all patients and adjunctive coiling was performed in 44(95.7%) patients. The rate of procedural-related complications was 13.0% (6/46), including 2 hemorrhagic and 4 ischemic complications. One patient died of rerupture of aneurysm (1/46, 2.2%), and 95.3% of patients (41/43) had favorable outcomes at the 90-day follow-up. Among 40 available cases, complete aneurysm occlusion was obtained in 38 cases (95.0%, 38/40) at a mean follow-up of 7.5 months.

**Conclusion:**

Treatment of ruptured complex IAs with the PED was associated with acceptable complication rates, high complete occlusion rates, and good clinical outcomes. Therefore, PED may be a safe and effective option for ruptured IAs that were difficult to treat by conventional endovascular and surgical approaches. However, larger and comparative studies with long-term follow-up are needed to confirm this result.

## 1 Introduction

Flow diverters (FDs) have been widely used to treat unruptured intracranial aneurysms (IAs), particularly those with challenging morphologies such as large or giant wide-neck, dissecting, or fusiform aneurysms (1–3). Among these FDs, the Pipeline Embolization Device (PED) has garnered significant attention, with many studies highlighting its safety and high efficacy (4–6).

However, the use of PED for acutely ruptured IAs remains controversial. The necessity of dual antiplatelet therapy (DAPT) to prevent stent thrombosis may increase the risk of hemorrhagic complications in subsequent invasive procedures, such as extraventricular drain (EVD) placement, craniotomy for decompression, or hematoma evacuation. Additionally, the risk of rebleeding after flow diversion is non-negligible, as the aneurysm is not immediately occluded.

Despite these limitations, the PED has been increasingly utilized to treat a subset of ruptured complex aneurysms that are difficult to manage with traditional clipping or coiling, and some studies have shown promising results (7, 8). However, the data remain limited. In this study, we report our experience with the Classical PED and PED Flex [45.7% (21/46) VS 54.3% (25/46)] in the treatment of acutely ruptured complex IAs. Our aim was to evaluate the safety, effectiveness, and follow-up outcomes of PED in the management of ruptured IAs.

## 2 Methods

### 2.1 Patients selection

This study included 46 consecutive patients with subarachnoid hemorrhage (SAH) secondary to ruptured complex aneurysms who were treated by PED placement (with or without adjunctive coiling) at our neurosurgical center between January 2019 and December 2023 (Supplementary Figure 1). Complex aneurysms were defined as aneurysms that were difficult to treat with conventional endovascular and surgical approaches, including blister-like aneurysms, dissecting aneurysms, fusiform aneurysms, large wide-neck saccular aneurysms (maximum diameter≥10 mm), and small wide-neck saccular aneurysms (diameter≤5 mm). Approval of the study protocol was obtained from the research ethics committee of our hospital, and all patients or their relatives had signed informed consent.

Patients were examined by non-contrast computed tomography (CT) to confirm SAH and the presence of hydrocephalus prior to the procedure. The need for EVD placement was assessed based on altered consciousness, ventricular dilatation, and hemorrhage size. If necessary, EVD was usually placed before loading the patient with DAPT.

### 2.2 Data collection and clinical assessment

For each patient, we recorded demographic data, initial neurological conditions, amount of SAH, morphology and location of the ruptured aneurysms, procedural details, clinical outcome, and follow-up angiographic results. The patient’s initial neurological condition was evaluated using World Federation of Neurosurgical Societies (WFNS) grading system, while the severity of SAH was classified according to the modified Fisher Scale (mFS).

### 2.3 Antiplatelet therapy

Patients received a loading dose of 300 mg aspirin and 300 mg clopidogrel orally or via nasogastric tube 4 h before the procedure. 5%Tirofiban (0.08ug/kg/min, Grandpharma Company, Wuhan, China) was pumped intravenously immediately after PED placement and continued for 24 h.

For patients without dual antiplatelet pretreatment, a bolus of tirofiban (5ug/kg, 5%Tirofiban) was injected slowly through the guiding catheter over a 3 min period immediately after PED deployment and a maintenance dose of 5% tirofiban (0.08 ug/kg/min) was pumped continuously for 24 h. A loading dose of 300 mg aspirin and 300 mg clopidogrel was conducted 4 h before the cessation of tirofiban.

On the next day, dual antiplatelet agents (100 mg aspirin and 75 mg clopidogrel) continued for 3 months and then, aspirin was used alone (100 mg daily) for 6 months. Aspirin and clopidogrel response testing was not mandatory.

### 2.4 Timing of endovascular treatment

Given that aneurysmal rebleeding constitutes a leading cause of mortality after SAH, occurring most frequently within the first 24 h, our center has adopted ultra-early treatment (≤24 h) as the goal of treatment.

### 2.5 Interventional procedure

**Figure 1:**
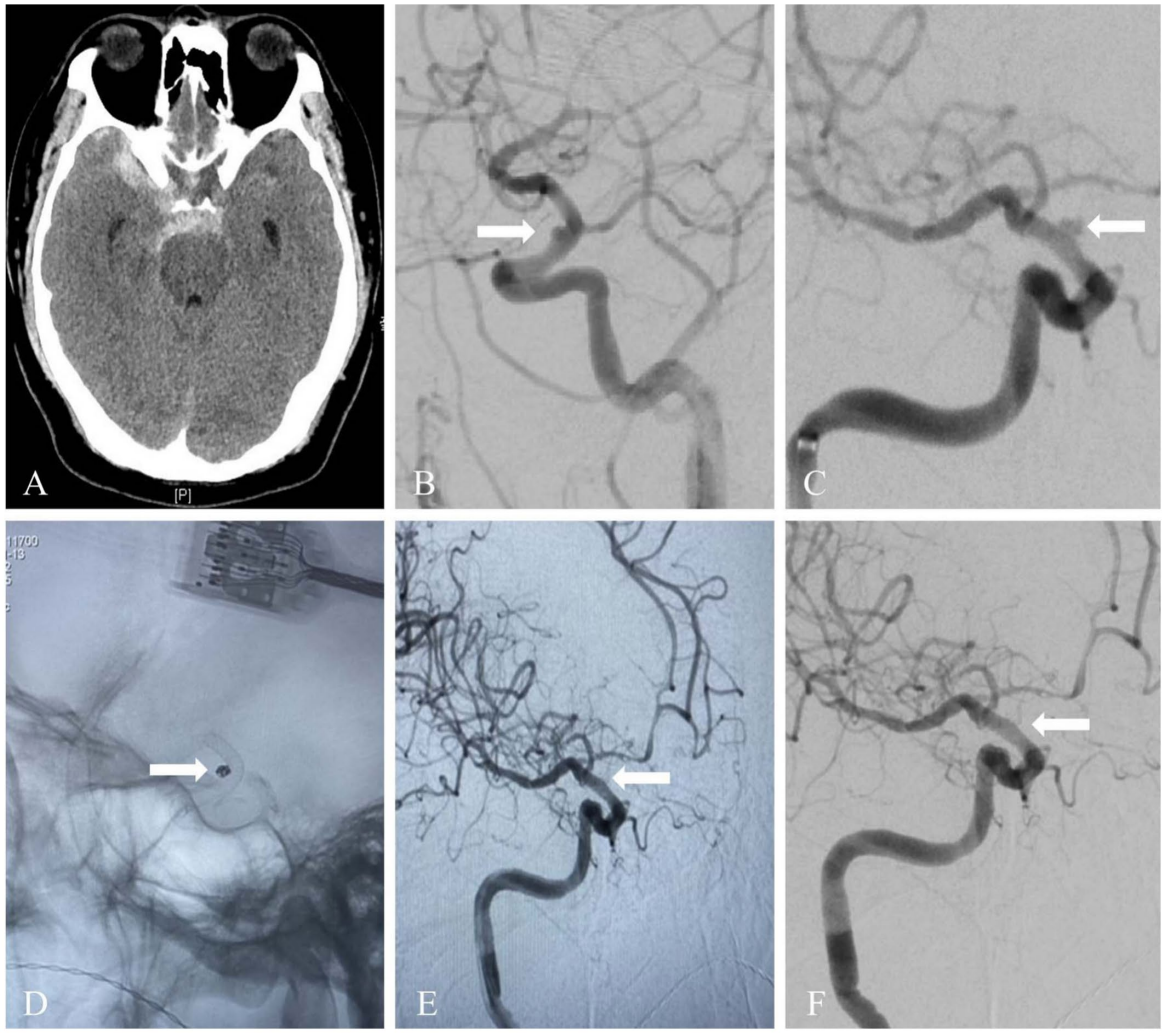
A ruptured blood blister aneurysm was treated with PED and adjunctive coiling. **(A)** Head computed tomography image of a 51-year-old patient showed acute subarachnoid hemorrhage. **(B,C)** Diagnostic cerebral angiography revealed a blood blister aneurysm located on the supraclinoid segment of the right internal carotid artery (white arrow). **(D)** The aneurysm was treated with PED and adjunctive coiling. Fluoroscopic image showed the flow diverter and coil mass (white arrow). **(E)** Immediate angiography after PED deployment showed complete occlusion (white arrow). **(F)** Follow-up angiography at 10 months showed complete occlusion of the aneurysm (white arrow).

**Figure 2:**
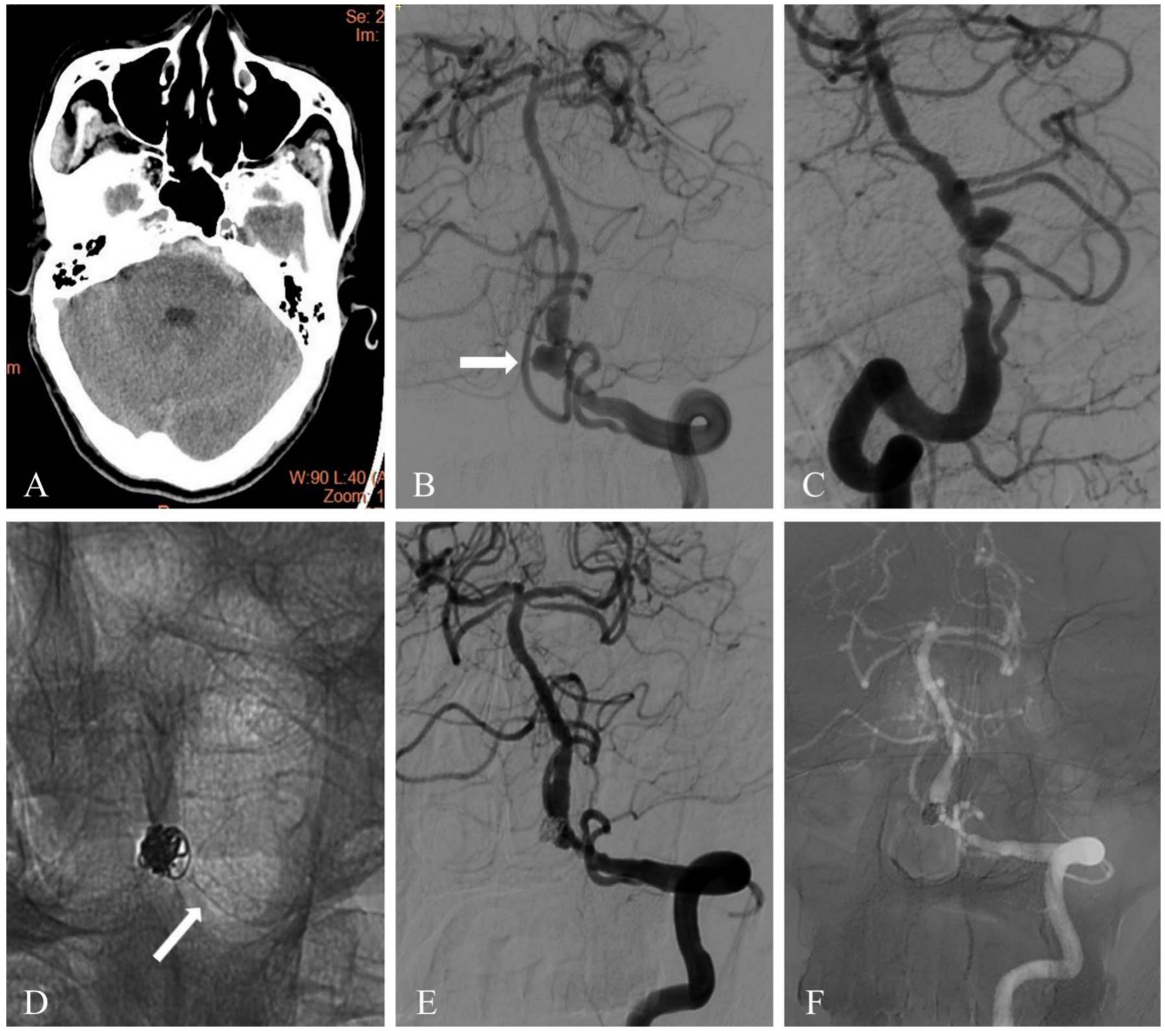
A 61-year-old patient presented with subarachnoid hemorrhage and a vertebrobasilar junction dissecting aneurysm. **(A)** Head computed tomography showed acute subarachnoid hemorrhage. **(B,C)** Diagnostic cerebral angiography revealed a dissecting aneurysm located in the vertebrobasilar junction (white arrow). **(D)** The dissecting aneurysm was treated with coiling and a PED. Unsubstracted angiogram showed the flow diverter (white arrow) and coil mass. **(E)** Immediate post-treatment angiography showed complete occlusion of aneurysm and patency of parent vessel. **(F)** Follow-up angiography at 8 months showed complete occlusion of aneurysm and parent vessel patency.

Endovascular procedures were performed through a right femoral artery approach using a short femoral 8-F sheath under general anesthesia. During the procedure, 3,000 IU heparin/500 mL saline was continuously dripped through the guiding catheter. An 8F Envoy guiding catheter (Cordis, United States) and 6F Navien (Medtronic, United States) intermediate catheter forming a coaxial system was inserted into target artery. The PED was deployed through a Marksman microcatheter (Medtronic, United States) or Phenom (Medtronic, USA) to cover the neck of the aneurysm. The diameter and length of the PED was assessed on angiography according to the size of the parent vessel. For aneurysms treated with additional coiling through a left transfemoral route using a 5-F femoral artery short sheath and a 5F Envoy guiding catheter (Cordis, United States), a microcatheter was placed inside the aneurysm dome, followed by partially deployment of PED to temporarily jail the microcatheter. Aneurysm coiling was subsequently performed and then the PED was completely deployed (Figures 1, 2).

### 2.6 Clinical and radiological follow-up

Clinical outcome was evaluated with modified Rankin Scale (mRS) scores (9). A favorable outcome was defined as a mRS score of 0 to 2 and a poor outcome was defined as a mRS score of 3 to 6 at 90 days after the procedure.

The angiographic follow-up was scheduled at 6 and 12 months with digital subtraction angiography (DSA). Aneurysm occlusions were evaluated according to the O’Kelly Marotta (OKM) grading scale: A, total filling (>95%); B, subtotal filling (5–95%); C, neck remnant (<5%); D, no filling (0%) (10). All clinical and imaging data were blindly measured by two independent clinicians who have 10 years of experience and any disagreements were resolved by a third physician with 15 years’ experience.

## 3 Results

### 3.1 Patients and aneurysm characteristics

A consecutive 46 patients with 52 aneurysms treated with PED were included in the study (Table 1). In 3 patients of multiple aneurysms, 6 neighboring unruptured aneurysms were also covered by the same PED. There were 16 males and 30 females, with a mean age of 55.8 ± 13.4 years (range 28-83 years).

**Table 1:** Patient and aneurysm characteristics.

Variables	Value
No. of patients	46
Age(years, mean ± SD)	55.8 ± 13.4
Sex, female, *n* (%)	30 (65.2)
WFNS grade, *n* (%)	
1	27 (58.7)
2	10 (21.7)
3	5 (10.9)
4	2 (4.3)
5	2 (4.3)
Fisher grade	
1	3 (6.5)
2	15 (32.6)
3	23 (50.0)
4	5 (10.9)
Type of aneurysm, *n* (%)	
Saccular	12 (26.1)
Blister	23 (50.0)
Dissecting	10 (21.7)
Fusiform	1 (2.2)
Aneurysm location, *n* (%)	
Middle cerebral artery (MCA)	3 (6.5)
Anterior communicating artery (ACOA)	1 (2.2)
Internal carotid artery (ICA)	33 (71.7)
Vertebral artery (VA)	7 (15.2)
Basilar artery (BA)	2 (4.3)
Aneurysm size [mean (range), mm]	4.3 (1.5–13.2)
Saccular	6.2 (4.2–13.1)
Blister	2.9 (1.5–4.9)
Dissecting	6.4 (5.3–11.8)
Fusiform	13.2
Treatment type, *n* (%)	
PED + coil	44 (95.7)
PED only	2 (4.3)

The ruptured aneurysms included 12 (26.1%) saccular, 23 (50.0%) blister-like, 10 (21.7%) dissecting, and 1(2.2%) fusiform. Thirty-seven ruptured aneurysms (80.4%) were located in the anterior circulation, and 9(19.6%) in the posterior circulation. Aneurysm size ranged from1.5 to 13.2 mm, with a mean of 4.3 ± 2.9 mm. If fusiform aneurysm was excluded (only one case), mean aneurysm size for saccular, blister, and dissecting aneurysms was 6.2 ± 3.0 mm (range4.2–13.1 mm), 2.9 ± 1.1 mm (range 1.5–4.9 mm), and 6.4 ± 3.5 mm (range 5.3–11.8), respectively. WFNS grades were I in 27 patients (58.7%), II in 10 (21.7%), III in 5 (10.9%), IV in 2 (4.3%), and V in 2 (4.3%). Eighteen patients (39.1%) had a lesser hemorrhage (mFS 1 or 2).

The deployment of PED was technically successful in all 46 patients and adjunctive coiling was used in 95.7% (44/46). The mean delay from bleed to PED placement was 1.8 days (range 0–4 days). Five patients underwent EVD before DAPT initiation, and none required VP shunt. A total of 49 Pipeline embolization devices were placed in 46 patients (mean 1.07 devices/patient; range 1–2).

### 3.2 Complications

As shown in Table 2, procedural-related clinical complications were recorded in 6 cases (2 hemorrhagic and 4 ischemic complications, 6/46, 13.0%) and resulted in permanent neurological morbidity in 2 (2/46, 4.3%) and death in 1(1/46, 2.2%).

**Table 2:** Procedure related complications.

Complications	Intra-procedural	Post-procedural	Total
Ischemic, *n* (%)			4 (8.7)
Transient	1 (2.2)	1 (2.2)	
Permanent	0	2 (4.3)	
Hemorrhagic, *n* (%)			
Rebleeding	0	1 (2.2)	2 (4.3)
Perforation	1 (2.2)	0	

Intraoperative aneurysmal rupture was observed in one case. A 37-year-old female patient presented with a ruptured wide-neck saccular aneurysm located in the left ophthalmic artery segment. The aneurysm was measured at 4.0 mm and 3.2 mm in width and height respectively, with neck size 3.9 mm. The patient was treated with PED Flex adjunctive coiling. During the procedure, the coil perforated the aneurysm wall and caused a small amount of SAH. The patient recovered well after immediate coiling. In another case, a patient with a dissection aneurysm of the anterior inferior cerebellar artery (AICA) was treated with PED Flex without adjunctive coiling. On the seventh day after treatment, the patient experienced rebleeding from the aneurysm and subsequently died.

During the procedure, in-stent thrombosis occurred in one patient and was immediately resolved with intravenous tirofiban. This patient suffered no symptoms. Additionally, three patients had an ischemic stroke after the procedure. One patient presented with a ruptured saccular aneurysm of the right posterior communicating artery (PCOM), accompanied by proximal stenosis of the parent vessel. Successful PED placement with partial coiling of the aneurysm was performed. Twenty-four hours post-procedure, the patient suffered from dysarthria. By immediately pumping tirofiban intravenously, the patient recovered well (mRS = 0). Another patient with a dissecting aneurysm, measured 10.1 mm × 5.8 mm in the M1 segment of left middle cerebral artery (MCA), was treated with PED Flex adjunctive coil emblization without complications. Two days postoperatively, the patient developed right-sided hemiparesis. The brain CT showed a left basal ganglia ischemia, which may be perforator infarction caused by PED implantation. The patient was managed with conservative treatment and had a permanent neurological deficit at discharge (mRS = 3). The third patient developed a small infarction on the second day following an uneventful PED adjunctive coil treatment for a large saccular aneurysm of the left PCOM. Platelet function testing revealed clopidogrel resistance, prompting us to switch to ticagrelor. The patient was discharged with an mRS score of 3.

### 3.3 Clinical and angiographic outcomes

As shown in Table 3, clinical follow-up data were available for 95.6% of patients (n = 43/45). Of the 43 patients, 41 (95.3%) had a good clinical outcome and 2 (4.7%) had a poor clinical outcome at the 90-day follow-up.

**Table 3:** Clinical and angiographic outcomes after flow diversion in ruptured aneurysms.

Outcomes	Value
Clinical follow-up, *n* (%)	43 (95.6)
Mean follow-up time (months)	3
Clinical outcome, *n* (%)	
mRS 0–2	41 (95.3)
mRS 3–6	2 (4.7)
Angiographic follow-up, *n* (%)	40 (88.9)
Mean follow-up time (months) [mean (range)]	7.5 (6–15)
Occlusion grade, *n* (%)	
Total filling (>95%)	0 (0.0)
Subtotal filling (5–95%)	2 (5.0)
Neck remnant (<5%)	0 (0.0)
No filling (0%)	38 (95.0)

Angiographic follow-up evaluations were available in 40(88.9%) of 45 surviving patients, with a mean follow-up time of 7.5 months (range 6–15 months). Grade D (no filling) was noted in 38/40 aneurysms (95.0%), Grade B (subtotal filling) was noted in 2 patients (5.0%, 2/40).

A mild degree of in-stent stenosis (<30%) was detected at 6-months follow-up angiography in one patient, which did not cause any symptoms.

### 3.4 Comparison between this study and preiovusly reported studies

Table 4 compared this study with previous studies (11–15). The current total complication rate (13.0%) and complete occlusion rate (95.0%) were similar to the results of recently published studies (11–15). The good clinical outcome rate was 95.3% in this study, higher than those of previous studies (11–15), which ranged from 68.9 to 86.3%. The rebleeding rate in our study was 2.2%, in the range from 0 to 5% of published case series (11–15).

**Table 4:** Comparison with previously reported studies.

Study	No. of Pts	Complications Ischemic hemorrhagic total (%) (%) (%)			Rebleeding rate (%)	Mean angiographic follow-up (months)	Complete occlusion rate (%)	mRS 0–2 (%)
Madaelil et al. (11)	126	5%	9%	12%	5%	6	90%	81%
Cagnazzo et al. (12)	223	8%	7%	17.8%	4%	9.6	89%	83%
Gopinathan et al. (13)	22	9%	4.5%	13.6%	0	NA	95%	86.3%
Almatter et al. (14)	45	11.1%	2.2%	13.3%	0	NA	94.6%	68.9%
Cohen et al. (15)	76	7.9%	0	7.9%	0	12	95%	81.6%
Current study	46	8.7%	4.3%	13.0%	2.2%	7.5	95%	95.3%

## 4 Discussion

In this study, we report our experience with the PED for acutely ruptured complex aneurysms, which are challenging to treat by endovascular coiling or clipping. In our series, the rebleeding rate was only 2.2% (1/46), and favorable clinical outcomes were 95.3% (41/43). At follow-up, complete occlusion was achieved in 95.0% (38/40). These results suggest that PED may be a safe and effective option for ruptured aneurysms that are considered difficult with conventional approaches.

However, the use of PED for ruptured IAs is controversial due to the necessity of DAPT and the delay in the aneurysm occluding, which may increase the risk of rebleeding. In the literature, the rebleeding rate was varied after FD deployment, ranging from 0 to 11% (11, 12, 15, 16). Cohen et al. (15) explored effective rebleeding protection of FD stents for acutely ruptured aneurysms. Their study included 76 patients and showed none of the patients had rebleed despite a very low rate of immediate complete exclusion. In a meta-analysis of 223 patients with ruptured aneurysms treated with FD stents, the aneurysm rebleeding rate was 4% (12). Similarly, in another meta-analysis, Madaelil et al. (11) reported aneurysm rerupture occurred in 5% of cases following FD placement. However, Ten Brinck et al. (16) conducted a retrospective study, which included 44 patients from 6 different centers, and found that the rate of rebleeding was relatively higher at 11%. In comparison, in our series, rerupture occurred in 2.2% of patients. The lower rebleed rate in our study was likely due to the fact that the majority of patients (95.7%, 44/46) had adjunctive coiling in the same session (17). Yang et al. (18) found that adjunctive use of coiling achieved higher incidence of immediate complete occlusion and no rebleed was encountered. Gopinathan et al. (13) reported their five-year experience and showed that none of the aneurysms rebled after flow-diversion and almost 73% (16/22) of patients were treated with adjunctive coiling.

Up to now, the optimal antiplatelet regimen for FD treatment in the acute rupture setting has no consensus (19). Aspirin plus clopidogrel was the most common agents used (7, 8, 20). However, because resistance to clopidogrel is commonly encountered, prasugrel and ticagrelor have also been used instead of clopidogrel in previous studies (15, 21, 22). In 2021, Gopinathan et al. (13) published a series of 22 patients with ruptured IAs treated with FDs. The patients received Ticagrelor(77%) and Prasugrel(15%) instead of clopidogrel as their antiplatelet regimen. Another alternative approach was that a maintenance dose of tirofiban was administered immediately after PED placement and continued for 12 h after the procedure (23–25). The advantages of tirofiban include its short half-life (2–4 h), rapid onset of action (about 5 min), and very potent inhibitory effect on platelets. The present study includes two different antiplatelet therapy regimens. In the first protocol, patients were loaded with 300 mg aspirin and 300 mg clopidogrel 4 h prior to the procedure. 5%Tirofiban (0.08 ug/kg/min) was administered intravenously immediately after PED placement and continued for 24 h. In the second protocol, patients without dual antiplatelet pretreatment were loaded with tirofiban (5ug/kg, 5%Tirofiban) slowly through the guiding catheter immediately after PED deployment and followed by a maintenance dose of tirofiban (0.08 ug/kg/min) for 24 h. A loading dose of 300 mg aspirin and 300 mg clopidogrel was overlapped with tirofiban infusion 4 h before the cessation of tirofiban. In our series, rebleeding was observed in only 1 patient (2.2%) and ischemic complications were record in 4 cases (8.7%). Our series suggests that the current antiplatelet protocol may be a viable option for patients with acute SAH. Recently, with the advancement of surface-modified technology, new FDs (such as PED-Shield, p48 MW and p64 MW HPC devices) have been used to treat acute ruptured aneurysms by single antiplatelet therapy (SAPT). Manning et al. (26) evaluated the safety and efficacy of PED-Shield for treatment of ruptured aneurysms with SAPT. They found no symptomatic ischaemic or haemorrhagic complications occurred in the patients without receiving post-operative heparin infusion. Lobsien D et al. (27) reported a 3-center experience with ruptured IAs treated with flow diverters with hydrophilic coating (p48 MW HPC and p64 MW HPC) under SATP. The follow-up imaging revealed all the stents were patent. Only one device-related thrombotic complication occurred. The COATING study by Laurent Pierot et al. (28) was the first prospective, multicenter, randomized controlled trial designed to compare the incidence of thromboembolic complications between patients treated with bare p64 MW under dual antiplatelet therapy (DAPT) and those treated with coated p64 MW HPC under single antiplatelet therapy (SAPT). The primary endpoint was the number of diffusion-weighted imaging lesions visualized via 3 T MRI within 48 h (±24 h) after the procedure. Preliminary outcome demonstrated no statistically significant difference between groups [2.1 (1.0, 4.0) vs. 2.5 (1.0, 5.0), *p* = 0.72], suggesting that drug-coated stents combined with SAPT are non-inferior to bare stents combined with DAPT for preventing perioperative thromboembolic events in unruptured or recurrent intracranial aneurysms. In the future, FDs with antithrombogenic properties may have greater prospects. However, clinical evidence of using such FDs in the treatment of acutely ruptured IAs is still lacking and large-sample prospective studies are needed.

In our series, the treatment-related complication rate was13.0% (6/46). A favorable clinical outcome was achieved in 95.3% of the patients and the rate of complete aneurysm occlusion was 95% at a mean 7.5-month follow up. These findings are aligned with previous studies. In a meta-analysis of 20 observational studies by Cagnazzo et al. (12), including 223 patients, the rate of complete/near-complete occlusion was nearly 90% associated with a complication rate of 18%. Good clinical outcome was achieved in 83% of the patients. Mahajan et al. (21) described a series of 16 SAH patients with Surpass FD treatment and reported 15 patients (94%) achieved favorable clinical outcome at 3 months. The complete occlusion rate on angiography was 87% (13/15) at 3 and 6 months and three patients (19%) developed transient hemiparesis. Gopinathan et al. (13) reported a similar rate of complete aneurysm occlusion achieved in 95%, with a good clinical outcome rate of 86.3%. Procedure- related adverse events were seen in 3 patients (13.64%). In our study, the high complete occulusion rate may be related to the following reasons. First, blister-like aneurysms account for 50% of the cases. In treating small-sized aneurysms, flow divert devices are more likely to cause high occlusion rate. Second, the combination of flow diverts with coiling is also a contributing factor to the high occlusion rate.

### 4.1 Limitations

Our study has several limitations, which should be considered while interpreting the results. First, it was a single-center retrospective study and thus there was inherited patient selection bias. Second, due to the small sample size and heterogeneity of aneurysms, we cannot make generalization about our results. Third, the follow-up period was short and a long-term follow-up is required. Despite the encouraging results in the present study, larger and comparative studies with long-term follow-up are needed to confirm its safety and efficacy.

## 5 Conclusion

Treatment of ruptured complex aneurysms with the PED was associated with acceptable complication rates, high complete occlusion rates, and good clinical outcomes. Therefore, PED may be a safe and effective option for ruptured complex IAs that were difficult to treat by conventional endovascular and surgical approaches. However, larger and comparative studies with long-term follow-up are needed to confirm this result.

## Data Availability

The datasets presented in this study can be found in online repositories. The names of the repository/repositories and accession number(s) can be found in the article/Supplementary material.
