# Supplementary material for: Utilization of pipeline embolization device for treatment of ruptured complex intracranial aneurysms
Source: Front Neurol. 2026 Jan 13;16:1673718. doi: 10.3389/fneur.2025.1673718 (PMC12834710; doi:10.3389/fneur.2025.1673718)
Supplement: Supplementary file 1 [file Data_Sheet_1.docx]

Supplemental figure 1: Flowchart of participant selection.


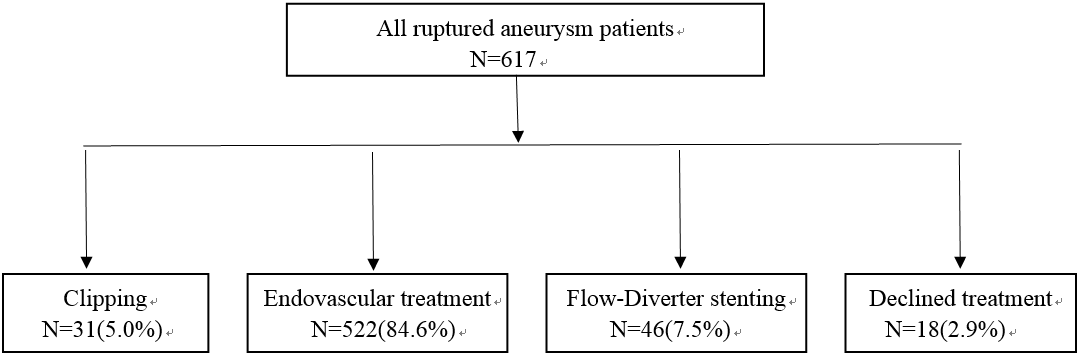


**Supplementary Table 1** Aneurysm characteristics

| Aneurysm type | Max diameter  mean (range) mm | Neck size  mean (range) mm | Dome-neck ratio  mean (range) |
| --- | --- | --- | --- |
| Saccular | 6.3(4.3-13.1) | 3.85(1.5-6.5) | 1.4(0.8-1.9) |
| Blister | 2.9(1.5-5.1) | 2.84(1.4-4.8) | 0.9(0.5-1.1) |
| Dissecting | 6.5(5.3-12.3) | NA | NA |
| Fusiform | 13.20 | NA | NA |
